# Supplementary material for: Lysophosphatidic acid as a CSF lipid in posthemorrhagic hydrocephalus that drives CSF accumulation via TRPV4-induced hyperactivation of NKCC1
Source: Fluids Barriers CNS. 2022 Sep 6;19:69. doi: 10.1186/s12987-022-00361-9 (PMC9450297; doi:10.1186/s12987-022-00361-9)
Supplement: Supplementary file 1 — Additional file 1. LPA quantification, choroid plexus viability, and 3D imaging of the rat ventricular system. [file 12987_2022_361_MOESM1_ESM.pdf]

A

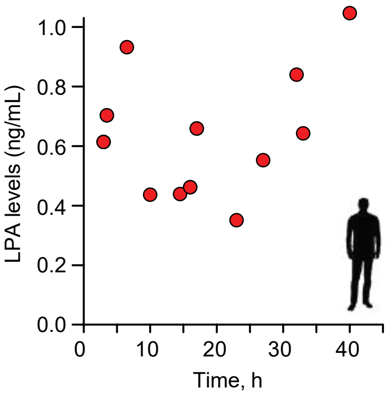

A No positive correlation between time from ictus to sampling and LPA levels in SAH patients
LPA levels (ng/ml) detected in CSF samples as a function of time from patients with SAH. Statistical evaluation with Pearson correlation coefficient. $R^2 = 0.03$; P = 0.57, n=12.

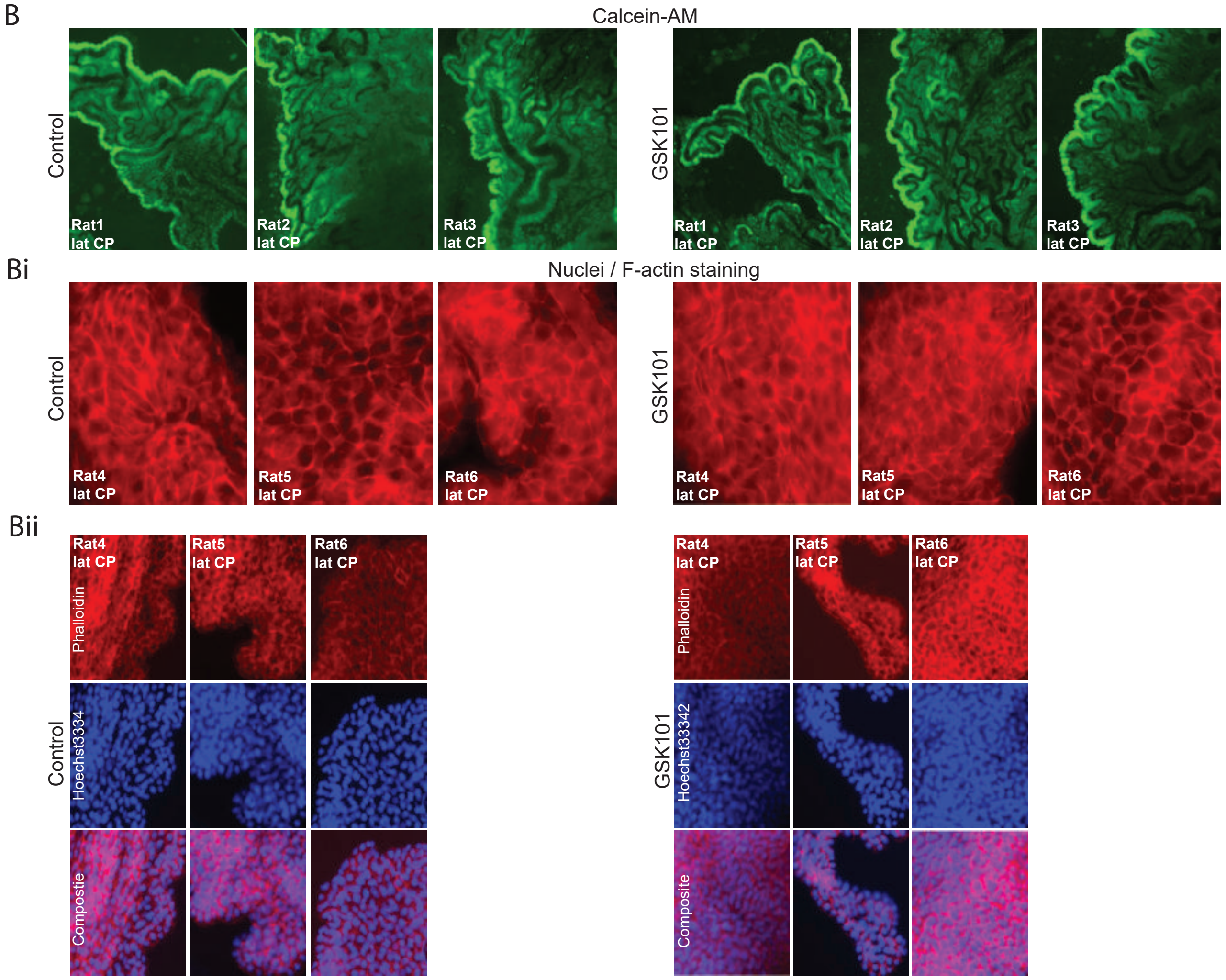

B Choroid plexus viability and cell integrity is not affected by 1h of GSK101 application
B Calcein acetoxymethyl (calcein-AM) [71] and **Bi-Bii** Hoechst/F-actin staining verified choroid plexus epithelial cell viability and maintained cell integrity. Lateral choroid plexuses was incubated in aCSF for 1h at 37°C with 500 nM GSK101. Non-treated choroid plexus served as control (VEH = DMSO). Following, the tissue was incubated in aCSF containing **B** calcein-AM (16.7 µM, C3100MP, Invitrogen) for 10 min at room temperature and transferred to microscope slides for microscopy or stained with **Bi-Bii** Hoechst33342 (62249, ThermoFisher) and Phalloidin555 (A34055, Invitrogen) for 20 min at room temperature and transferred to microscope slides for microscopy (mounted with ProLong Gold Antifade Reagent, P10144, ThermoFisher). Images were acquired using Zeiss Axioplan 2 microscope equipped with epifluorescence and interference filters with 702 moni AxioCam (Zeiss Zen Black software). n=3.

C

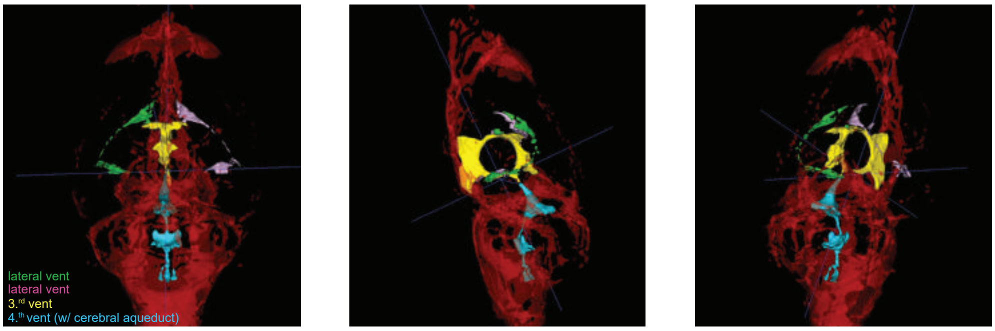

C 3D rendering of ventricular CSF space in rat

3D structure of the ventricular CSF space from magnetic resonance imaging used to measure the volume of the ventricular system. Lateral ventricles shown in green and pink, 3rd ventricle shown in yellow and 4th ventricle (with the cerebral aqueduct) shown in turquoise.
